# Association between malaria and household air pollution interventions in a predominantly rural area of Ghana

**DOI:** 10.1186/s12936-022-04431-z

**Published:** 2023-03-23

**Authors:** Kwaku Poku Asante, Blair J. Wylie, Felix B. Oppong, Ashlinn Quinn, Stephaney Gyaase, Alison G. Lee, Kenneth Ayuurebobi Ae-Ngibise, Katrin Burkart, Ellen Abrafi Boamah-Kaali, Seyram Kaali, Steven Chillrud, Patrick L. Kinney, Seth Owusu-Agyei, Darby Jack

**Affiliations:** 1 Kintampo Health Research Centre, Research and Development Division, Ghana Health Service, Kintampo North Municipality, Bono East Region, Ghana; 2 Department of Obstetrics and Gynecology PH Building, Columbia University Medical Center, 16th Floor 622 West 168th Street, New York, NY 10032 USA; 3 Berkeley Air Monitoring Group, 1935 Addison St., Suite A, Berkeley, CA 94704 USA; 4 Division of Pulmonary, Critical Care and Sleep Medicine, Icahn School of Medicine at Mount Sinai, New York, NY USA; 5 Institute for Health Metrics and Evaluation, University of Washington, 2301 Fifth Ave., Seattle, WA 98121 USA; 6 Lamont-Doherty Earth Observatory at Columbia University, Palisades, NY USA; 7 Department of Environmental Health, Boston University School of Public Health, Boston, MA USA; 8 Institute of Health Research, University of Health and Allied Sciences, Ho, Ghana; 9 Department of Environmental Health Sciences, Mailman School of Public Health at Columbia University, 722 W 168th Street, New York, NY 10032 USA

## Abstract

**Background:**

Though anecdotal evidence suggests that smoke from HAP has a repellent effect on mosquitoes, very little work has been done to assess the effect of biomass smoke on malaria infection. The study, therefore, sought to investigate the hypothesis that interventions to reduce household biomass smoke may have an unintended consequence of increasing placental malaria or increase malaria infection in the first year of life.

**Methods:**

This provides evidence from a randomized controlled trial among 1414 maternal-infant pairs in the Kintampo North and Kintampo South administrative areas of Ghana. Logistic regression was used to assess the association between study intervention assignment (LPG, Biolite or control) and placental malaria. Finally, an extended Cox model was used to assess the association between study interventions and all episodes of malaria parasitaemia in the first year of infant’s life.

**Results:**

The prevalence of placental malaria was 24.6%. Out of this, 20.8% were acute infections, 18.7% chronic infections and 60.5% past infections. The study found no statistical significant association between the study interventions and all types of placental malaria (OR = 0.88; 95% CI 0.59–1.30). Of the 1165 infants, 44.6% experienced at least one episode of malaria parasitaemia in the first year of life. The incidence of first and/or only episode of malaria parasitaemia was however found to be similar among the study arms.

**Conclusion:**

The findings suggest that cookstove interventions for pregnant women and infants, when combined with additional malaria prevention strategies, do not lead to an increased risk of malaria among pregnant women and infants.

## Background

Malaria remains a disease of public health importance and poses a significant burden on people living in sub-Saharan Africa (SSA), the region with the heaviest malaria burden. Although malaria infection is widespread among people living in malaria endemic areas, pregnant women and children under 5 years are most affected [1]. Malaria infection is four times higher among pregnant women compared with non-pregnant women, and malaria mortality is two times higher in pregnant women [2]. Among pregnant women in SSA, malaria has been found to be associated with anaemia, spontaneous abortion, preterm delivery, low birth weight and perinatal mortality [3]. Infants born to mothers with placental malaria are more susceptible to malaria infection and anaemia in early life [4–8].

In Ghana, malaria remains burdensome particularly among pregnant women and children. The prevalence of malaria parasitaemia is about 20% among pregnant women at the national scale [9]. Out of the over 1.5 million malaria-related hospital admissions recorded in 2016, 46.7% were among children under five years. Likewise, 43.7% of the 30,332 total malaria deaths recorded in Ghana in 2016 occurred among children less than five years [10]. Malaria predominantly affects rural dwellers [11, 12], where biomass fuel use such as firewood and charcoal for cooking is also high [13–15]. In 2020, biomass fuel use account for about 35% of the total energy used in Ghana [16].

It has been suggested previously that smoke from household biomass fuel use reduces malaria risk through a reduction in human biting by the malaria vector, female *Anopheles gambiae* [1]. To date, although anecdotal evidence suggests that smoke from HAP has a repellent effect on mosquitoes, very little work has been done to assess the effect of biomass smoke on malaria infection. Seven previous epidemiologic studies have focused on the association between cooking fuel use and malaria. Two studies found positive associations between biomass fuel use and malaria, one found a negative association and four had null findings [17–25]. A 2007 literature review addressing the relationship between household air pollution and malaria risk concluded that no evidence supports the hypothesis that biomass smoke reduces malaria risk. The authors of that review further argue that the benefits of biomass smoke exposure reduction far outweigh any possible increase in malaria risk [26].

Leveraging on the Ghana Randomized Air Pollution and Health Study (GRAPHS), this study investigated the hypothesis that, interventions to reduce household biomass smoke may have an unintended consequence of increasing placental malaria or increasing malaria in the first year of life. This provides the first evidence from a randomized controlled trial regarding a possible relationship between biomass smoke and malaria risk.

## Methods

### Study area

GRAPHS was conducted among 1414 maternal-infant pairs in the Kintampo North and Kintampo South administrative areas of Ghana, West Africa. The area is largely rural and are monitored by an extensive health and demographic surveillance system. Neonatal and infant mortality is 32 deaths per 1000 live births and 52 deaths per 1000 live births [27]. The inhabitants are mostly farmers and depend primarily on biomass fuel for their cooking needs. In an earlier study involving 421 households, biomass fuel use was recorded among 99% of the households [29].

### Study design

This study was conducted as part of the Ghana Randomized Air Pollution and Health Study (GRAPHS) which has been described in detail elsewhere [28] and on clinical trials.gov (Trial Registration NCT01335490). In brief, GRAPHS was a cluster randomized intervention trial designed to determine the impact of clean cooking interventions on birth weight and pneumonia in the first year of life. The burden of malaria is high in the study area [30]. Pregnant women in intervention clusters received either a two-burner liquefied petroleum gas (LPG) stove along with LPG, which was supplied to them throughout the study duration or two improved biomass stoves (BioLite^™^, Brooklyn, New York). Pregnant women in control clusters did not receive any of the intervention, but used their traditional biomass three-stone fire. All pregnant women and children were provided insecticide-treated nets (ITNs) for use throughout the study.

### Recruitment of study participants and study procedure

Pregnant women from 35 communities were identified via community-based fieldworkers [28], taking advantage of the existing Kintampo Health and Demographic Surveillance System [27]. Women who were confirmed by ultrasound to be carrying a single live foetus of ≤ 24 weeks estimated gestation and who gave consent to participate in the trial were enrolled. Inclusion criteria also required participants to be non-smokers and the primary cook in their households. Details of the study procedure have been reported elsewhere [28]. Study women were followed throughout pregnancy until delivery.

### Exposure measurement

Smoke produced from cooking with carbon-based fuels is an indication of incomplete combustion which results in a complex mixture of particulate matter and gases. Carbon monoxide (CO) is one of the incomplete combustion products for which there existed viable, miniaturized, low cost sensors available at the time of the GRAPHSs study. As such, small, lightweight CO monitors (Lascar EL-CO-USB Data Logger, Essex, UK) were used for personal sampling among all women and infants in the study at defined time points. An initial exposure baseline assessment was done immediately after enrollment and prior to delivery of the clean stove intervention. Three additional exposure monitoring sessions were completed, initially approximately three weeks after initiation of the cooking intervention, and at two additional time points evenly spaced across the remaining expected weeks of gestation. Women and their infants both underwent postnatal exposure monitoring at months one, three and nine, for a total of 7 exposure sessions on each enrolled woman and three on each infant.

At each measurement occasion, a series of 72-h personal CO exposure assessment were made. Using the Lascar monitors, CO was recorded every ten seconds in parts per million (ppm). The detection limit of the Lascar’s 10 s readings is ca. 3 ppm. In a typical indoor/outdoor household setting, the unit read zero much of the time. These readings were however not replaced since it was assumed that true exposure was likely to be close to zero when no combustion events were occurring. The averages over the multiday monitoring sessions were driven by brief time periods, typically around meal times, with very high CO concentrations (10 s to > 1000 ppm). After data cleaning, each deployment was truncated to the first 48 h to avoid having an uneven number of cooking events captured for units that did not run for the entire 72 h. Using a time-weighted average approach, prenatal average CO from the beginning of pregnancy to birth and a postnatal average CO from birth to the first birthday of the child was computed from the individual 48 h averages.

### Placental sample collection and processing

Births occurred in the communities and at health facilities. Fieldworkers lived within the communities in the study area and operated 24-h surveillance to identify home deliveries and also collect placental tissues. Pregnant women were encouraged to deliver in the health facilities, but were advised to send for the community-based fieldworker when they were in labour if they decided to deliver at home with a trained or untrained birth attendant’s assistance. In the health facilities, fieldworkers also ran 24-h surveillance to identify and collect placental tissues from participants who delivered at these facilities. Participants were identified by the fieldworkers at the maternity wards by direct questioning and by observing the participants maternity card which had been labelled at the time of recruitment or during the pregnancy follow up visits.

Whole placenta was collected together with the umbilical cord when it was expelled from the uterus by the attending nurse or birth attendant during the third phase of labour. It was placed flat in a basin with the maternal surface (irregular surface opposite the umbilical cord insertion) up. A full thickness placental biopsy (2.5 × 2.5 × 2.5 Cm^3^) was taken from maternal surface halfway between the placental edge and the umbilical cord insertion by a trained fieldworker, the attending nurse or birth attendant. Gross macroscopic abnormalities such as infarcts (deep red or whitish areas of firm consistency in acute and old infarcts, respectively); massive fibrin deposition (whitish area of firm consistency) and placental cyst were avoided during the biopsy process. The biopsy was placed in a pre-labelled container of a 50 ml of 10% buffered formalin. It was transported to the Seth Owusu-Agyei Medical Laboratory at KHRC by a field supervisor for storage at room temperature until further processing for histology. A maximum of four (4) hours was allowed between placental expulsion and placental biopsy to avoid placental necrosis.

### Placental histology slide preparation

Placental histology slides were prepared at the Pathology Department of University of Ghana Medical School in Accra, Ghana. Placental tissues were processed by placing it in labelled Tissue Tek (Sakura Finetek USA Inc., USA) cassettes and fixed in 10% buffered formalin for 1.5 h. The tissues were dehydrated and cleared in two changes of xylene and infiltrated in molten paraffin wax at melting point of 56 °C. The tissues were embedded in the molten paraffin wax in cassettes to form tissue blocks. The tissue blocks were trimmed of excess wax and cut into thin sections using a microtome. The sections were fixed onto a microscope slide, de-waxed in three changes of xylene and hydrated in descending grades of ethanol and then transferred through 3 changes of absolute ethanol. The sections were then stained in haematoxylin and eosin, cleared in xylene for 5 min and mounted with DPX mountant. Using the methods described by Bulmer et al. [8, 31], a histopathologist determined the status of placental sample as not infected (no parasites or pigment present); acute infection (parasites present, and pigment deposition within fibrin or cells within fibrin minimal or absent), chronic infection (parasites present, and pigment deposition in fibrin or cells within fibrin substantial); or past infection (parasites absent but pigment present).

After delivery, infants were followed for 12 months. Community fieldworkers repeatedly visited participating households to check on the health of infant study participants and ill infants were referred to a study clinician for care. All illnesses were managed according to the Ghana National Treatment Guidelines. Where indicated, thick and thin blood smears were prepared to make a diagnosis of malaria parasitaemia as described by Swysen et al. [32].

### Covariates

At enrolment, data on demographic, socio-economic and obstetric characteristics of study participants were collected by trained fieldworkers using standard questionnaires. These include data on maternal age, gravidity (first pregnancy vs. other) and marital status. Also, an overall wealth index was computed for each study participant using principal component analysis [33, 34]. Study participants were grouped by their wealth indices into wealth quintiles namely, least poor, less poor, poor, more poor, and most poor. After delivery, data on intermittent preventive treatment in pregnancy using sulfadoxine-pyrimethamine (IPTp-SP), maternal use of ITNs, malaria transmission season at delivery and child sex were also obtained using a questionnaire.

### Statistical analysis

Cleaned data were analysed using Stata version 14.0 (Stata-Corp, College Station, TX). Univariate and multiple logistic regression were used to assess the association between study intervention assignment (LPG, Biolite or control) and placental malaria. Likewise, logistic regression was used for the associations between maternal prenatal CO exposure and placental malaria. The analysis was adjusted for maternal age, wealth index, gravidity (first pregnancy), IPTp, use of ITNs, marital status, and malaria transmission season at delivery, factors known to be associated with placental malaria.

Cox regression was used to determine the hazard of first or only episode of malaria parasitaemia between the control, Biolite and LPG arms. Kaplan–Meier plot for the probability of infants in the control, Biolite and LPG arms living without malaria was provided. An extended Cox model for multiple/recurrent events—Andersen-Gill model [35] was used for the association between study interventions and all episodes of malaria parasitaemia in the first year of infant’s life. Robust standard errors were used to account for the within-child correlation. Maternal age, child sex, wealth index, first pregnancy, IPTp, marital status, placental malaria and malaria transmission season at delivery were included as possible confounding variables. Similarly, an extended Cox model with robust standard errors was used for the association between infant CO exposure in the first year of life and all episodes of malaria parasitaemia in the first year of life.

## Results

### Characteristic of study participants

A total of 1414 pregnant women were enrolled in the GRAPHS study. Placental tissue was collected for 1193 (84.4%) of the total study mothers. Those not collected included mothers who refused for their samples to be taken, births that occurred outside the study area and instances where fieldworkers were not notified of the birth. Study mothers whose placental tissues were not collected as well as children of these mothers were excluded from this analysis. Of the 1193 participants who were included in this analysis, 427 (35.8%) were from the control arm, 438 (36.7%) from Biolite arm and 328 (27.5%) from the LPG arm. The average age of study participants was about 28 years (SD = 7.2). Overall, 15.0% of the study participants were carrying their first pregnancies. With regards to malaria prevention interventions, 857 (71.84%) of the study participants received at least one dose of IPTp and 764 (64.04%) used ITNs during pregnancy. A total of 28 infant deaths (2.4%) were recorded for the 1193-study participant and the remaining 1165 infants were followed for one year for malaria parasitaemia. There was almost an equal distribution of sex among the infants born to the study participants: 49.6% female vs 50.4% males. More than two thirds (67.3%) of the infants were born during the high malaria transmission season (Table 1).

**Table 1:** Characteristics of study participants by study arm

Variable	Total cohort	Three stone	Biolite	LPG
Household characteristics	(N = 1193)	(n = 427)	(n = 438)	(n = 328)
Wealth index; n (%)				
Least poor	234 (19.61)	103 (24.12)	87 (19.86)	44 (13.41)
Less poor	236 (19.78)	82 (19.20)	91 (20.78)	63 (19.21)
Poor	240 (20.12)	57 (13.35)	113 (25.80)	70 (21.34)
More poor	246 (20.62)	91 (21.31)	87 (19.86)	68 (20.73)
Most poor	237 (19.87)	94 (22.01)	60 (13.70)	83 (25.30)
Home ownership				
Yes	631 (52.89)	192 (44.96)	243 (55.48)	196 (59.76)
Maternal characteristics	(N = 1193)	(n = 427)	(n = 438)	(n = 328)
Age; mean (SD)	27.99 (7.17)	28.21 (7.60)	28.46 (7.13)	27.09 (6.56)
First pregnancy; n (%)				
Yes	179 (15.00)	66 (15.46)	57 (13.01)	56 (17.07)
No	1014 (85.00)	361 84.54)	381 (86.99)	272 (82.93)
IPTp use; n (%)				
Yes	857 (71.84)	320 (74.94)	308 (70.32)	229 (69.82)
No	336 (28.16)	107 (25.06)	130 (29.68)	99 (30.18)
Use of ITNs; n (%)				
Yes	764 (64.04)	239 (55.97)	290 (66.21)	235 (71.65)
No	429 (35.96)	188 (44.03)	148 (33.79)	93 (28.35)
Marital status				
Married	669 (56.08)	236 (55.27)	256 (58.45)	177 (53.96)
Living together	376 (31.52)	155 (36.30)	134 (30.59)	87 (26.52)
Single	144 (12.07)	33 (7.73)	47 (10.73)	64 (19.51)
Widowed/Divorced	4 (1.05)	3 (1.47)	1 (0.23)	0 (0.00)
Prenatal CO exposure				
Participants with data	1182 (99.08)	425 (99.53)	435 (99.32)	322 (98.17)
CO/ppm (continuous); mean (SD)	1.32 (1.17)	1.44 (1.32)	1.34 (1.22)	1.15 (0.81)
Infant characteristics	(N = 1165)	(n = 417)	(n = 428)	N = (320)
Sex of baby				
Female	578 (49.61)	220 (52.76)	191 (44.63)	167 (52.19)
Male	587 (50.39)	197 (47.24)	237 (55.37)	153 (47.81)
Malaria transmission season at delivery; n (%)				
High	784 (67.30)	291 (69.78)	278 (64.95)	215 (67.19)
Low	381 (32.70)	126 (30.22)	150 (35.05)	105 (32.81)
Postnatal CO exposure				
Participants with data	1048 (89.96)	374 (89.69)	385 (89.95)	289 (90.31)
CO/ppm (continuous); mean (SD)	0.94 (1.73)	0.91 (1.20)	1.02 (2.09)	0.88 (1.78)

### Associations between study interventions and placental malaria

The prevalence of placental malaria was 24.6%, of which 20.8% were acute infections, 18.7% chronic infections and 60.5% past infections. There was no significant association between the study interventions and all types of placental malaria (Table 2). In both the unadjusted and adjusted analysis, compared to the control participants, the odds of placental malaria were similar for Biolite and LPG participants when compared to participants in the control arm. Maternal prenatal CO exposure was not significantly associated with placental malaria (Table 2).

**Table 2:** Association between assigned cook stove, prenatal CO exposure and placental malaria

Variable	Placental malaria		Unadjusted OR (95% CI)	p-value	Adjusted OR (95% CI)	p-value
	Yes (N = 294) n (%)	No (N = 899) n (%)				
Study intervention						
Control stove	103 (24.12)	324 (75.88)	referent		referent	
Biolite	105 (23.97)	333 (76.03)	0.99 (0.73–1.35)	0.959	1.01(0.71–1.45)	0.942
LPG	86 (26.22)	242 (73.78)	1.12 (0.80–1.56)	0.510	0.88 (0.59–1.30)	0.521
Prenatal CO/ppm exposure						
Participants with data	289 (98.29)	893 (99.33)	0.94 (0.83–1.07)	0.359	1.00 (0.87–1.15)	0.965

The adjusted analysis includes maternal age, wealth index, nulliparity, and receipt of IPTp, use of ITNs, marital status, and malaria transmission season at delivery

### Malaria parasitaemia among infants

#### Associations between study interventions and malaria parasitaemia in infants

Out of the 1165 infants, 520 (44.6%) experienced at least 1 episode of malaria parasitaemia in the first year of life. In all, 923 distinct episodes of malaria (ranging from one to eight per child) were experienced over the twelve-month period of follow-up. Only one infant whose mother was assigned to the control group did not experience any form of malaria throughout the period of follow-up. On the average, infants whose mothers were assigned to the control and Biolite stove experienced approximately the same number of distinct episodes during the follow-up, though the maximum distinct episodes was experienced by infants whose mothers were assigned to the Biolite stove (Table 3). The incidence of first and/or only episode of malaria parasitaemia was similar among the study arms (control, Biolite and LPG). Likewise, there were no significant differences in the incidence of all episodes of malaria parasitaemia in the first year of life for the control, Biolite and LPG users (Table 4).

**Table 3:** Summary of placental malaria and episodes of malaria over the 12 months follow up by intervention

Intervention	Placental malaria		Number of episodes	Average episodes	Minimum	Maximum
	Yes (N = 284) n (%)	No (N = 881) n (%)				
Control stove	98 (34.5)	319 (36.2)	339	2.6	0	6
Biolite	102 (35.9)	326 (37.0)	348	2.5	1	8
LPG	84 (29.6)	236 (26.8)	237	2.3	1	6

**Table 4:** Incidence of malaria parasitaemia in infants according to the intervention of their mother

Intervention	No	Person–years	Incidence rate per infant per year (95% CI)
First or only episode of malaria			
Three-stone	183	228.2	0.80 (0.69–0.93)
Biolite	198	226.3	0.88 (0.76–1.01)
LPG	139	184.2	0.75 (0.64–0.89)
Total (overall)	520	638.7	0.81 (0.75–0.89)
All episodes of malaria in the first 12 months			
Three-stone	338	297.9	1.13 (1.02–1.26)
Biolite	348	307.5	1.13 (1.02–1.26)
LPG	237	242.4	0.98 (0.86–1.11)
Total (overall)	923	847.8	1.09 (1.02–1.16)

**Fig. 1:**
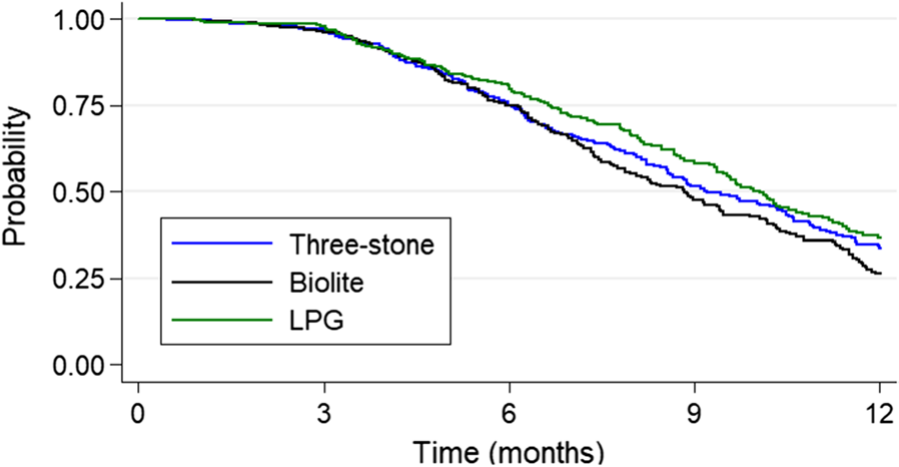
Kaplan–Meier curves for the risk of first or only episode of malaria distinguished by intervention type

In both adjusted and unadjusted analyses, the hazard of first and/or only episode of malaria parasitaemia was similar in the LPG and Biolite arms compared to the control arm. The infant’s exposure to CO was also not statistically associated with first and all episodes of malaria parasitaemia in the first year of life (Table 5). This finding is further illustrated using a Kaplan–Meier plot (Fig. 1).

**Table 5:** Association between study intervention, infant CO exposure and hazard for first/only episode and all episodes of malaria parasitaemia in the first year of life

Variable	Unadjusted analysis		Adjusted analysis	
	Hazard Ratio (95% CI)	p-value	Hazard Ratio (95% CI)	p-value
First/only episode of malaria parasitemia				
Study intervention				
Three-stone	Referent		Referent	
Biolite	1.09 (0.89–1.34)	0.357	1.05 (0.84–1.29)	0.676
LPG	0.89(0.72–1.11)	0.315	0.89 (0.71–1.13)	0.351
Infant CO/ppm exposure	1.01 (0.95–1.08)	0.666	1.00 (0.94–1.07)	0.916
All episodes of malaria parasitemia				
Study intervention				
Three-stone	Referent		Referent	
Biolite	1.00 (0.82–1.22)	0.974	0.94 (0.76–1.16)	0.572
LPG	0.82 (0.66–1.02)	0.082	0.80 (0.63–1.01)	0.059
Infant CO/ppm exposure	1.00 (0.97–1.04)	0.796	1.00 (0.97–1.04)	0.811

The adjusted analysis includes maternal age, child’s sex, wealth index, nulliparity, receipt of IPTp, maternal marital status, malaria transmission season at delivery, and placental malaria

## Discussion

This study sought to investigate the hypothesis that interventions to reduce household biomass smoke may have unintended risks of increasing placental malaria and/or malaria in the first year of life. There was no significant increased risk of placental malaria nor infant malaria resulting from interventions that reduced exposure to biomass smoke. The findings from this study is consistent with findings in a retrospective case controls study conducted in Burkina Faso among pregnant women and children less than 9 years of age [21].

Several factors may explain the equal risk of malaria among the invention. It is likely that the biting times of malaria vectors occur at different times from household cooking times. Female *Anopheles* are known to be primarily active late at night and bite indoor when most cooking activities would have ended and community members likely to be indoors [36, 37]. Secondly, in the study area, households frequently use efficient malaria control tools or have equal malaria risk from other exposures such as poor housing infrastructure. In this cohort, ITN use and use of intermittent preventive treatment of malaria during pregnancy was documented to be high among both interventions as well as control groups and may have equally reduced the burden of malaria. Housing architecture with presence of eaves may present a higher risk of malaria. Within the study area, majority of houses have eaves that allow mosquitoes enter the houses at night putting women and children at risk of malaria. Furthermore, the intervention targeted individual pregnant women and not entire communities. The findings in this analysis of exposure data, found that the effect of the intervention did not adequately reduce the household biomass smoke exposure as they could be exposed to smoke from neighbouring households and other indiscriminate burnings in the communities. For instance, households in the LPG study arm living in a densely populated area had average 48-h CO exposure of 1.00 ppm (SD: 2.58), whereas those living less dense area had average 48-h CO exposure of 0.72 ppm (SD: 0.88) [38].

The relatively low average CO exposures in GRAPHS compared to other studies on household cooking are consistent with majority of the cohort cooking outdoors or in semi enclosed settings. Different results could be possible in settings where cooking occurs in poorly vented indoor locations where the smoke maybe more of a deterrent to mosquitoes.

## Conclusion

The findings suggest that cookstove interventions for pregnant women and infants, when combined with additional malaria prevention strategies, do not lead to an increased risk of malaria among pregnant women and infants. Thus, concerns about increasing malaria risk should not impede transitions to clean household energy.

## Data Availability

The data will be made available upon reasonable request to the corresponding author.

## References

[1] WHO. World Malaria Report 2022. Geneva: World Health Organization; 2022. ISBN 978-92-4-006489-8. Assessed on 20 Mar 2023. https://www.who.int/publications/i/item/9789240064898

[2] Groves J, El-Shirbiny D (2015). Revision notes for the DRCOG: a textbook of women’s health.

[3] Desai M, ter Kuile FO, Nosten F, McGready R, Asamoa K, Brabin B (2007). Epidemiology and burden of malaria in pregnancy. Lancet Infect Dis.

[4] Borgella S, Fievet N, Huynh B-T, Ibitokou S, Hounguevou G, Affedjou J (2013). Impact of pregnancy-associated malaria on infant malaria infection in southern Benin. PLoS ONE.

[5] Bardají A, Sigauque B, Sanz S, Maixenchs M, Ordi J, Aponte JJ (2011). Impact of malaria at the end of pregnancy on infant mortality and morbidity. J Infect Dis.

[6] Le Hesran JY, Cot M, Personne P, Fievet N, Dubois B, Beyeme M (1997). Maternal placental infection with *Plasmodium falciparum* and malaria morbidity during the first 2 years of life. Am J Epidemiol.

[7] Le Port A, Watier L, Cottrell G, Ouédraogo S, Dechavanne C, Pierrat C (2011). Infections in infants during the first 12 months of life: role of placental malaria and environmental factors. PLoS ONE.

[8] Ismail MR, Ordi J, Menendez C, Ventura PJ, Aponte JJ, Kahigwa E (2000). Placental pathology in malaria: a histological, immunohistochemical, and quantitative study. Hum Pathol.

[9] Dosoo DK, Chandramohan D, Atibilla D, Oppong FB, Ankrah L, Kayan K (2020). Epidemiology of malaria among pregnant women during their first antenatal clinic visit in the middle belt of Ghana: a cross sectional study. Malar J.

[10] National Malaria Control Programme. 2017 Annual Report. Accra, Ghana, 2017.

[11] Robert V, Macintyre K, Keating J, Trape J-F, Duchemin J-B, Warren M (2003). Malaria transmission in urban sub-Saharan Africa. Am J Trop Med Hyg.

[12] Keiser J, Utzinger J, De Castro MC, Smith TA, Tanner M, Singer BH (2004). Urbanization in sub-saharan Africa and implication for malaria control. Am J Trop Med Hyg.

[13] GSS: 2010 population and housing census: summary report of final results. Ghana Statistical Service Accra; 2012.

[14] Donnelly MJ, McCall P, Lengeler C, Bates I, D'Alessandro U, Barnish G (2005). Malaria and urbanization in sub-Saharan Africa. Malar J.

[15] Ghana Statistical Service. 2010 Population and housing census: summary report of final results. Accra, Ghana, 2012.

[16] Energy Commission (2021). National Energy statistics 2020.

[17] WHO (2008). Smoke and malaria: are interventions to reduce exposure to indoor air pollution likely to increase exposure to mosquitoes and malaria?.

[18] Bockarie M, Service M, Barnish G, Momoh W, Salia F (1994). The effect of woodsmoke on the feeding and resting behaviour of *Anopheles gambiae* s.s.. Acta Trop..

[19] Ndibazza J (2012). Spatial distribution and risk factors of malaria in infancy in Entebbe. Uganda Trop Med Int Health.

[20] Semakula HM, Song G, Zhang S, Achuu SP (2015). Potential of household environmental resources and practices in eliminating residual malaria transmission: a case study of Tanzania, Burundi. Malawi and Liberia Afr Health Sci.

[21] Yamamoto S, Louis V, Sie A, Sauerborn R (2010). Household risk factors for clinical malaria in a semi-urban area of Burkina Faso: a case–control study. Trans R Soc Trop Med Hyg.

[22] Snow R, Bradley A, Hayes R, Byass P, Greenwood B (1987). Does woodsmoke protect against malaria?. Ann Trop Med Parasitol.

[23] Danis-Lozano R, Rodriguez M, Gonzalez-Ceron L, Hernandez-Avila M (1999). Risk factors for *Plasmodium vivax* infection in the Lacandon forest, southern Mexico. Epidemiol Infect.

[24] Wisit CD (2005). Behavioral factors and malaria infection among the migrant population, Chiang Rai Province. J Med Assoc Thailand.

[25] Yamamoto S, Sauerborn R, Sié A (2007). Biomass smoke: does it offer protection against severe malaria?. Epidemiology.

[26] Biran A (2007). Smoke and malaria: interventions to reduce exposure to indoor air pollution likely to increase exposure to mosquitoes?. Trans R Soc Trop Med Hyg.

[27] Owusu-Agyei S, Nettey OE, Zandoh C, Sulemana A, Adda R, Amenga-Etego S (2012). Demographic patterns and trends in Central Ghana: baseline indicators from the Kintampo Health and Demographic Surveillance System. Glob Health Action.

[28] Jack DW, Asante KP, Wylie BJ, Chillrud SN, Whyatt RM, Quinn AK (2015). Ghana randomized air pollution and health study (GRAPHS): study protocol for a randomized controlled trial. Trials.

[29] Van Vliet ED, Asante K, Jack DW, Kinney PL, Whyatt RM, Chillrud SN (2013). Personal exposures to fine particulate matter and black carbon in households cooking with biomass fuels in rural Ghana. Environ Res.

[30] Dery DB, Brown C, Asante KP, Adams M, Dosoo D, Amenga-Etego S (2010). Patterns and seasonality of malaria transmission in the forest-savannah transitional zones of Ghana. Malar J.

[31] Bulmer JN, Rasheed FN, Francis N, Morrison L, Greenwood BM (1993). Placental malaria. I. Pathological classification. Histopathology.

[32] Swysen C, Vekemans J, Bruls M, Oyakhirome S, Drakeley C, Kremsner P (2011). Development of standardized laboratory methods and quality processes for a phase III study of the RTS, S/AS01 candidate malaria vaccine. Malar J.

[33] Asante KP, Owusu-Agyei S, Cairns M, Dodoo D, Boamah EA, Gyasi R (2013). Placental malaria and the risk of malaria in infants in a high malaria transmission area in Ghana: a prospective cohort study. J Infect Dis.

[34] Vyas S, Kumaranayake L (2006). Constructing socio-economic status indices: how to use principal components analysis. Health Policy Plan.

[35] Andersen PK, Gill RD (1982). Cox’s regression model for counting processes: a large sample study. Ann Statist.

[36] Bayoh MN, Walker ED, Kosgei J, Ombok M, Olang GB, Githeko AK (2014). Persistently high estimates of late night, indoor exposure to malaria vectors despite high coverage of insecticide treated nets. Parasit Vectors.

[37] Tuno N, Kjaerandsen J, Badu K, Kruppa T (2010). Blood-feeding behavior of *Anopheles gambiae* and *Anopheles melas* in Ghana, western Africa. J Med Entomol.

[38] Chillrud SN, Ayuurebobi K, Gould CF, Owusu-Agyei S, Mujtaba M, Manu G (2021). The effect of clean cooking interventions on mother and child personal exposure to air pollution: results from the Ghana Randomized Air Pollution and Health Study (GRAPHS). J Expo Sci Environ Epidemiol.

